# Adaptive Evolution of the OAS Gene Family Provides New Insights into the Antiviral Ability of Laurasiatherian Mammals

**DOI:** 10.3390/ani13020209

**Published:** 2023-01-06

**Authors:** Gang Liu, Xiaoyang Wu, Yongquan Shang, Xibao Wang, Shengyang Zhou, Honghai Zhang

**Affiliations:** College of Life Sciences, Qufu Normal University, Qufu 273165, China

**Keywords:** Laurasiatherian mammals, evolution, OAS gene family, antiviral

## Abstract

**Simple Summary:**

As the dividing line between human and wild mammal habitats becomes smaller, virus invasion due to frequent environmental changes poses a significant risk to many mammals. It is becoming increasingly important to research the antiviral ability of wild mammals. As an antiviral gene family, the OAS gene family plays an important role in resistance to viruses. As an important group of mammals, Laurasiatherian mammals occupy a variety of ecological niches in nature and play a crucial role in maintaining the stability of the ecosystem. Our findings offer insights into the molecular and functional evolution of the antiviral ability of Laurasian mammals by exploring the adaptive evolution of the OAS gene family of Laurasiatherian mammals.

**Abstract:**

Many mammals risk damage from virus invasion due to frequent environmental changes. The oligoadenylate synthesis (OAS) gene family, which is an important component of the immune system, provides an essential response to the antiviral activities of interferons by regulating immune signal pathways. However, little is known about the evolutionary characteristics of OASs in Laurasiatherian mammals. Here, we examined the evolution of the OAS genes in 64 mammals to explore the accompanying molecular mechanisms of the antiviral ability of Laurasiatherian mammals living in different environments. We found that *OAS2* and *OAS3* were found to be pseudogenes in Odontoceti species. This may be related to the fact that they live in water. Some *Antilopinae*, *Caprinae*, and *Cervidae* species lacked the *OASL* gene, which may be related to their habitats being at higher altitudes. The OASs had a high number of positive selection sites in *Cetartiodactyla*, which drove the expression of strong antiviral ability. The OAS gene family evolved in Laurasiatherian mammals at different rates and was highly correlated with the species’ antiviral ability. The gene evolution rate in *Cetartiodactyla* was significantly higher than that in the other orders. Compared to other species of the Carnivora family, the higher selection pressure on the OAS gene and the absence of positive selection sites in Canidae may be responsible for its weak resistance to rabies virus. The OAS gene family was relatively conserved during evolution. Conserved genes are able to provide better maintenance of gene function. The rate of gene evolution and the number of positively selected sites combine to influence the resistance of a species to viruses. The positive selection sites demonstrate the adaptive evolution of the OAS gene family to the environment. Adaptive evolution combined with conserved gene function improves resistance to viruses. Our findings offer insights into the molecular and functional evolution of the antiviral ability of Laurasian mammals.

## 1. Introduction

The OAS gene family is induced by type I, type II and type III interferons (IFNs) [1], and its products are activated by double-stranded viral RNA (dsRNA) [2,3]. IFNs play an important role in animals’ immune systems. Many genes can be activated to resist virus invasion under the action of IFNs. Interferons are divided into three classes, type I interferons (IFNα/β), type II interferons (IFNγ) and the more recently discovered type III interferons (IFNλ), all of which are defined by discrete receptor complexes [4]. Type I interferons are a large class of interferons that are responsible for the host immune response to viral and bacterial infections [5]. Types I and III are mainly produced immediately after viral infection, while type II is produced by secondary activated T lymphocytes and NK cells [6].

The OAS gene family contains four genes: *OAS1*, *OAS2*, *OAS3* and *OAS-Like (OASL*). All OAS members have a highly conserved N-terminal OAS domain that plays a key role in preventing viral infection [1]. *OAS1*, *OAS2* and *OAS3* have 2′-5′oligoadenylate synthase activities and exert antiviral activities through a classical RNase L-dependent pathway [7]. *OASL* acts as a multifaceted immunomodulator to adjust antiviral activities [8]. The *OAS1* gene has one catalytic domain and forms a tetramer, the *OAS2* gene has two catalytic domains and forms a dimer, and the *OAS3* gene has three catalytic domains and is a monomer. 2′-5′ oligomers with different lengths are synthesized by OAS genes that occupy different subcellular locations [9]. Mammalian 2′-5′ oligo adenylate synthases (2′-5′-OASs) are essential enzymes in interferon-induced antiviral responses. They catalyze the polymerization of ATP into 2′-5′ linked oligo adenylates, which activate the constitutively expressed latent endonuclease RNaseL, thereby blocking viral replication at the level of mRNA degradation [10]. Some scholars have suggested that gene duplications and domain fusion events result in paralogs that provide another means of escaping pathogen inhibitors [11]. OASs also play an important role in the fight against enteroviruses [12]. Furthermore, there is also evidence of the concerted adaptive evolution of *OASs* and STING [13]. In addition to having antiviral functions, OAS proteins are involved in other cellular events, including gene induction, apoptosis, cell growth and differentiation [14].

Laurasiatherian mammals comprise six orders, namely, *Eulipotyphla*, *Chiroptera*, *Cetartiodactyla*, *Pholidota*, *Perissodactyla* and *Carnivora* [15]. Laurasiatherian species occupy a wide range of ecological niches in nature, which increases their chances of becoming infected by a wide range of viruses. Laurasiatherian mammals have also been the focus of viral research. Among them, *Chiroptera* [16] and *Carnivora* [17] are the most investigated animals in the current research on virus transport and transmission. Some scholars have found that the eigenvector centrality measure of all virus species is the largest in wild animal species (*Carnivora*, *Chiroptera*, *Artiodactyla* and *Primates*). This means that the importance of a node depends on both the number of its neighboring nodes and the importance of its neighboring nodes. This means that virus species are most abundant in wildlife due to the combined comparison of multiple hosts of virus distribution. However, RNA viruses make up a relatively large proportion of virus species infecting *Carnivora* and *Chiropteran* rather than DNA viruses [17]. Scientists have demonstrated that bats carry significantly higher levels of viruses than other mammals [18]. Several domesticated ungulates (*Cetartiodactyla* and *Perissodactyla*) are outliers in observed virus numbers. Virus abundance in these species has been found to be higher than in other species, but these species have relatively low rates of zoonotic viruses [18]. Laurasiatherian mammal species also have a place in cancer research; studies have identified several copy number amplifications in cetaceans associated with immunity, aging and cancer, suggesting that cetaceans are among the large mammals that have evolved specific adaptations associated with cancer resistance [19].

According to some scholars, the local extinction of wild animals is largely due to the large-scale outbreak of diseases caused by viruses [20]. As a typical RNA virus, rabies virus (RABV) is the most common causative agent of rabies, and it is distributed in almost all countries of the world and can infect a wide range of mammal species [21]. Documented host species of RABV include domestic animals and wild-living carnivores, comprising foxes and raccoon dogs in Europe [22]; foxes in the Middle East; raccoon dogs and ferret-badgers in Asia; skunks [23], foxes, coyotes and mongooses in the Americas; and African civet and mongooses in Africa [24]. Bats also carry a very high number of rabies viruses as hosts for many viruses [25,26].

At present, there are few studies focusing on the association between the evolutionary characteristics of OAS genes and the of Laurasiatherian species. We explored the evolutionary and developmental relationships of the OAS gene family in Laurasiatherian species to investigate the effect of OAS gene family on the antiviral ability of animals.

## 2. Materials and Methods

### 2.1. Genome Dataset Preparation and Sequence Alignments

Our dataset encompassed Laurasiatherian mammal lineages: *Cetartiodactyla* (*n* = 21), *Pholidota* (*n* = 2), *Perissodactyla* (*n* = 4), *Carnivora* (*n* = 26), *Chiroptera* (*n* = 5) and *Eulipotyphla* (*n* = 6). All genomes involved in this study were obtained from the National Center for Biotechnology Information (NCBI: accessed on 15 November 2021 https://www.ncbi.nlm.nih.gov/). These species were selected on the basis of their niche and lifestyle. NCBI is an excellent tool to study the evolution of species based on the molecular data available and to reveal certain phenomena of species quickly and efficiently [27]. Detailed information on Laurasiatherian species and relevant information on the genomes are listed in Table S1.

We used an online Web BLAST search and the NCBI database to find annotated OAS genes. Following the online web search, we directly downloaded the sequenced OAS genes by selecting one or two species under each order. We downloaded the protein-coding sequences (CDSs) of the species. We used these sequences as query sequences to investigate the genomes of unannotated Laurasiatherian mammals. For each target genome, we created a local database and used the BLASTN algorithm [28] and BLASTn in TBtools v1.0 [29] to search for OAS-encoding sequences. According to the E-value cutoff of 10^−5^, we confirmed whether the screening sequence matches the genome significantly by picking the higher scoring fragments. Following that, target sequences were obtained using the GeneWise website (accessed on 7 March 2022 https://www.ebi.ac.uk/Tools/psa/genewise/). Finally, we employed MEGA X (v7.0.14) [30] software to determine whether the protein-coding sequence is interrupted by a stop codon and to delete the stop codon.

### 2.2. Molecular Evolutionary Analyses

We performed a phylogenetic analysis on the screened OAS gene families. The target nucleotide sequences of the Laurasiatherian mammals were converted to amino acid sequences using MEGAX software. MUSCLE’s Log-Expectation software was used to compare the transformed amino acid sequences (Multiple Sequence Comparison by Log-Expectation) [31]. IQ-Tree software [32] was used to build a maximum likelihood (ML) tree with 1000 bootstrap replications. We examined the most suitable nucleotide substitution model using ModelTest and the Bayesian criterion (BIC) [33]. We eventually identified GTR + T as the most suitable model. The phylogenetic tree was edited with Interactive Tree of Life (iTOL) [34]: an online tool for phylogenetic tree display and annotation [35]. The substitutions of nonsynonymous (dN) and synonymous (dS) genes were calculated and compared using Codeml in PAML(v4.9) [36]. Positive Darwinian selection pressure on genes is typically determined by calculating the nonsynonymous (dN)/synonymous (dS) substitution ratio (ω) between homologous protein-coding gene sequences. Ω (ω) > 1, < 1, = 1 represent positive selection, negative selection and neutral evolution, respectively [37]. Based on the sequence alignments and the phylogenetic trees downloaded from TimeTree (http://www.timetree.org/ (accessed on 15 June 2022)), we carried out the selective force imposed on Laurasiatherian mammals’ OAS genes using a codon-based codeml PAML 4.9 program. In the selection pressure analysis, we used the same selection pressure parameters for all OAS genes. We performed a selection pressure analysis of the OAS genes for each order, and multiple corrections were made for LRT and *p*-values [38].

To determine the signatures of natural selection on the OAS genes in Laurasiatherian mammals, we used the site model in CodeML to explain the different evolution rates and changes in functionality by amino acid site [39]. The site model allows ω values to vary across sites in the same sequence. We used the M7 (null hypothesis: 0 < ω < 1) and M8 (alternative hypothesis: ω > 1) models to test for positive selection loci of the OAS gene. The selection of the accepted models was based on the model likelihood ratio test (LRT) results. If the likelihood test *p*-value was significant, the alternative hypothesis M8 model was accepted (positive selection model). For meaningful models, the Bayes Empirical Bayes (BEB) method can be used to detect positively selected sites. A potential positive selection site is identified when the posterior probability is greater than 0.9 [40]. We also performed a positive selection site analysis using the Fast Unconstrained Bayesian AppRoximation (FUBAR) model in Datamonkey (http://www.datamonkey.org/ (accessed on 10 December 2022)) [41,42].

Next, we used the branch model to test the overall evolutionary characteristics in all branches. In this model, the positive selection model MA (Model = 2, NSsites = 2) is compared with the null model (Fix_ω = 1, ω = 1). The former is set to mean that the ω values of all clades in the phylogenetic tree vary; the latter is set to mean that the ω values of all clades are equal. The branch model is used to test the adaptive evolution of OAS genes across species clades [43]. In order to investigate whether only some sites are under positive selection along the foreground branches, we used the branch-site model and set each family as foreground branches to detect the effect of positive selection on some sites [44]. We compared the null model (Fix_omega = 1, Omega = 1) with the positive selection model MA (Model = 2, NSsites = 2). The posterior probability of a positively selected locus detected in the significant model was calculated using an empirical Bayesian approach. Loci were considered to have undergone positive selection when the posterior probability value was greater than 0.90.

## 3. Results

### 3.1. OAS Gene Identification and Gene Tree Reconstruction

**Figure 1:**
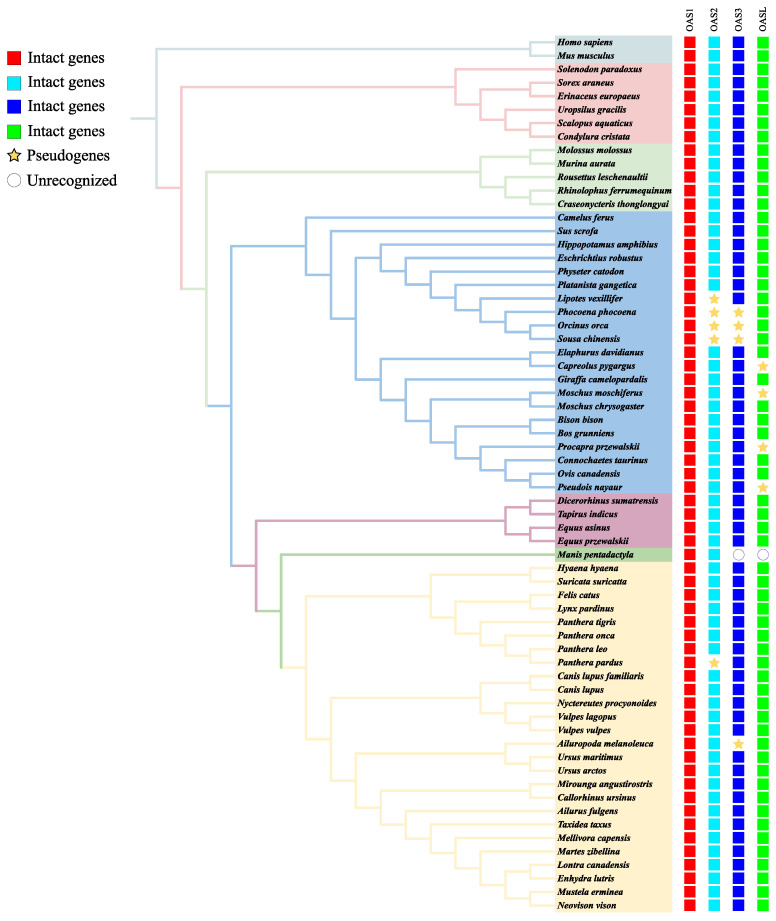
Species tree for the animals used in this study and the intact OAS gene number in these animals.

**Figure 2:**
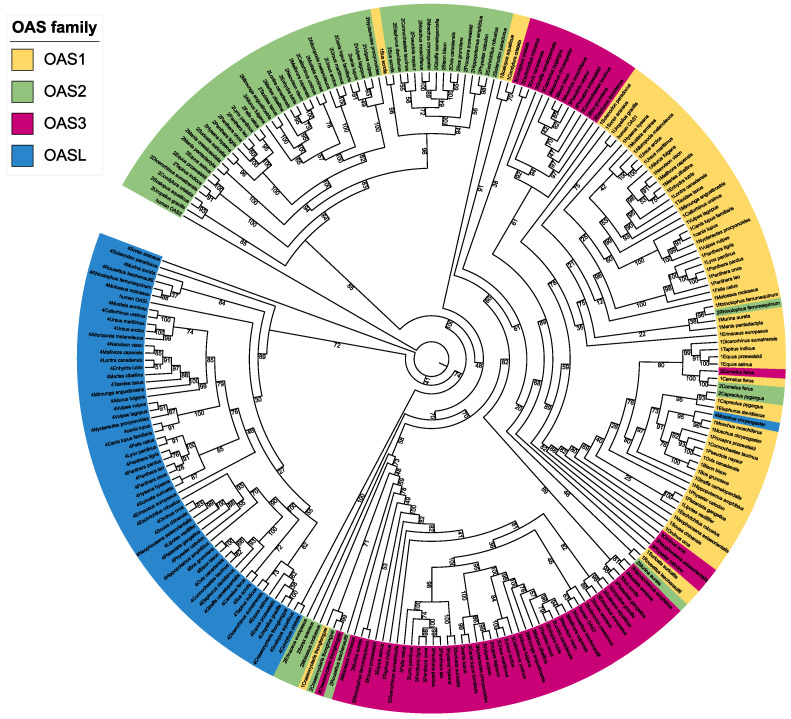
Phylogenetic tree of the OAS genes in Laurasiatherian mammals.

We obtained 250 genes related to OAS in 64 mammalian species, namely 238 intact genes and 12 pseudogenes, and 6 genes that were not identified in the two Pholidota species. We also added the human *OAS1* and *OAS2* genes as outgroups. Figure 1 shows the results of the download from Timetree according to the selected species, with different branch colors according to the family that the species belongs to and the addition of a genetic screen (Table S1 and Figure 1). The phylogenetic tree of these genes is similar to the relative distribution of the species tree (Figure 1 and Figure 2). We found that the *OASL* gene was relatively independent compared to other genes and did not have massive duplication with other genes in the Laurasiatherian mammals. This also shows that *OASL* is different from the other genes in the OAS family. The duplication of genes occurs in a number of species. The numbers in Figure 1 are standard bootstrap values, which indicate the confidence level of branch formation.

### 3.2. Selection Characteristics of OAS Genes

**Figure 3:**
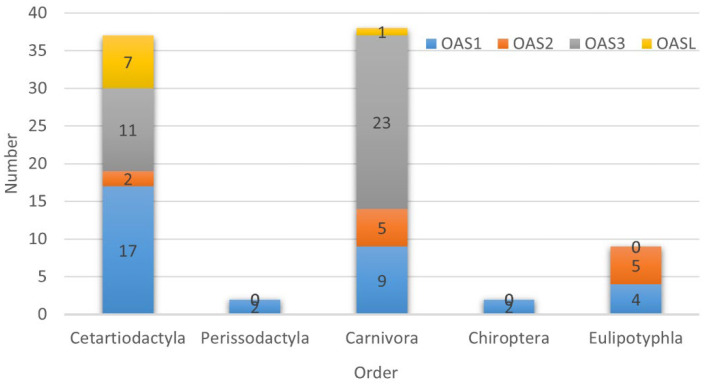
Analysis of positive selection sites for Laurasiatherian mammal species.

In the test of positive selection using the site model, we identified positive selection sites in each gene of *Cetartiodactyla*, indicating that these genes experienced positive selection during the process of species evolution. A total of 37 positive selection sites were detected in the following four genes: *OAS1* had 17 positive selection sites (BEB > 0.95), 8 of which were extremely significant (BEB > 0.99); *OAS2* had 2 positive selection sites; *OAS3* had 11 positive selection sites (BEB > 0.95), 7 of which were extremely significant (BEB > 0.99); and *OASL* had 7 positive selection sites (BEB > 0.95), 3 of which were extremely significant (BEB > 0.99). Simultaneously, the *p*-values for the four genes were extremely significant (*p* < 0.01). We identified a significant positive selection site in the OAS1 gene of *Carnivora.* We identified 37 positive selection sites in the OAS gene of *Cetartiodactyla*, namely 18 extremely significant and 19 significant sites. We identified two positive selection sites in the *OAS1* gene in both *Perissodactyla* and *Chiroptera*. We identified 9 significant positive selection sites in *Eulipotyphla*. We identified 37 positive selection sites in *Carnivora*, namely 19 significant sites and 18 extremely significant sites. No positive selection sites were found in *Pholidota* (Figure 3 and Table 1). We used the FUBAR model in Datamonkey for an analysis of the positive selection sites (Table 2), which was discussed for each order. The results show that Carnivora had the lowest number of positive selection sites, while Cetartiodactyla had the most positive selection sites.

We used the branch model to examine the selection pressure for OAS genes in the Laurasiatherian mammals to determine whether adaptive evolution occurred in a specific branch. The results of the branching model analysis showed that the ω values of OAS genes ranged from 0.114–1.034. It is worth noting that the evolution rates of the *OASL* gene in *Cervidae* and *Giraffidae* in *Cetartiodactyla* were subject to positive selection. Other than that, all other OAS genes are under the pressure of purifying selection [45] (Table 3).

**Figure 4:**
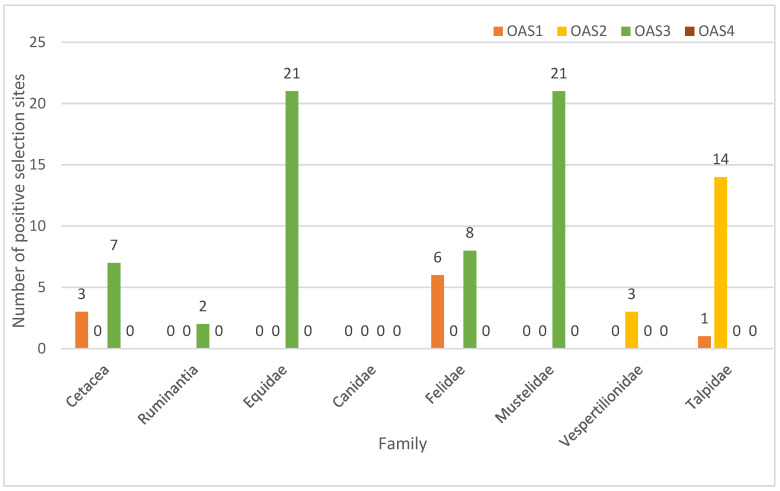
Positive selection for OAS gene branch locus models in Laurasiatherian mammals.

Each order of Laurasiatherian mammals plays an extremely important ecological role in nature. Each of these orders are relatively easily influenced by a variety of RNA viruses. *Cetartiodactyla* and *Carnivora* occupy more diverse ecological niches. In order to determine whether positive selection occurred in each type, we selected well-characterized taxa from each order as foreground branches. The specific results of the branch site model are in Table S2. We divided *Cetartiodactyla* into *Cetacea* and *Ruminantia*. In order to explore the evolutionary pressures on the OAS gene family in different survival environments, we identified positive selection sites in the *OAS1* and *OAS3* genes of *Cetacea*. Meanwhile, positive selection sites were identified in the *OAS3* gene of *Ruminantia* [46]. In *Carnivora*, we selected *Canidae*, *Felidae* and *Mustelidae* as the foreground branches [47]. We found positive selection sites in *OAS1* and *OAS3* for Felidae and in *OAS3* for *Mustelidae*. No positive selection sites were found in Canidae. We chose Equidae as a foreground branch because of the small number of *Perissodactyla* species, and we found a large number of positive selection sites in *OAS3*. Positive selection sites were found in *OAS3* and *OASL* of *Vespertilionidae*. Positive selection sites were found in *OAS1* and *OAS2* in *Talpidae* (Figure 4 and Table S2).

## 4. Discussion

The OAS gene is well-known as an antiviral gene, and the antiviral effects of *OAS1*, *OAS2* and *OAS3* are dependent on the classic OAS/RNase L signaling pathway [7]. *OASL* acts as a multifaceted immunomodulator to promote host antiviral responses by enhancing the interaction of tMDA5 and tMAVS, even though *OASL* lacks the enzymatic activity to synthesize 2′-5′ oligoadenylate [9,48]. It is well-known that the loss or pseudogenization of any gene may have a significant impact on various abilities [49,50,51]. Interestingly, we discovered that several species’ OAS genes contained pseudogenes. In *Cetartiodactyla*, the *OAS2* gene and *OAS3* gene were found to be missing in *Neophocaena asiaeorientalis*, *Sousa chinensis*, *Lipotes vexillifer* and *Orcinus orca*. A possible explanation for this might be that, as they are involved in adaptation to aquatic life, the *OAS2* and *OAS3* genes were lost in *Cetacean*. At the same time, previous research has suggested that some other genes lost in *Cetaceans* were probably involved in adaptation to the lifestyle of all aquatic life [52]. This further supports the notion that the loss of ancestral genes may be a mechanism of phenotypic adaptation [53,54]. At the same time, some researchers have discovered that all cetaceans and star-nosed moles have a deletion of the RNA virus receptor gene DDX58, which could lead to the degradation of their antiviral genes [55]. Some *Cervidae* species (*Capreolus pygargus*, *Procapra przewalskii*, *Moschus moschiferus* and *Pseudois nayaur*) lacked *OASL*, which may make them vulnerable to viruses. However, these species have been found to be less susceptible to viral infections [18]. This confirms that the lack of the *OASL* gene leads to the enhanced expression of other OAS genes [56]. Moreover, genomic assemblies of too-poor quality or errors can occur with gene deletions and pseudogenes. The presence or absence of these genes in these species should be verified via PCR at a later stage.

The results of the phylogenetic analysis show that the OAS gene family is more clearly differentiated. The *OASL* gene can be clearly distinguished from other genes (Figure 2). This is similar to the results of previous studies [57]. At the same time, we found an interesting phenomenon: in several species, OAS gene duplication had occurred. The gene duplication phenomenon was found to have occurred mainly in *Cetartiodactyla*. The *OAS3* genes of *Bovidae*, *Moschidae* and *Cervidae* were clustered in one branch and were homologous to *OAS1.* The *OAS3* genes of *Orcinus orca*, *Physeter catodon* and *Neophocaena asiaeorientalis* were clustered into a single branch and were homologous to *OAS1*. The *OAS2* gene of *Capreolus pygargus* was homologous to *OAS1*. The *OAS2* and *OAS3* genes of *Camelus ferus* were clustered with the *OAS1* gene into a single branch. *OASL* and *OAS1* of *Moschus chrysogasterd* were clustered into one branch. *Sus scrofa*’s *OAS1* and *OAS2* were clustered into one branch. There are various reasons for such phenomena, such as possible duplication and loss, gene conversion or possibly the misidentification of genes leading to these results. We screened for similar species based on the CDS of the OAS gene; if the gene was missing, then no sequence would be obtained. If the genes were misidentified, then all closely related species genes would be in error, and we did not find this to be the case. Thus, the reason could only be gene duplication or gene conversion. But, to the best of our knowledge, we are not aware of any studies of gene conversion in the OAS gene. The present results provide some support for the idea that gene duplication is a general evolutionary mechanism in the OAS gene family [58]. There is no similar phenomenon in the OAS gene family of *Carnivora*. To the best of our knowledge, *Ruminantia* is not sensitive to RNA viruses among Laurasiatherian species, *Talpidae* is moderately susceptible to RNA viruses, *Chiroptera* is relatively susceptible to RNA viruses, and *Carnivora* is the most susceptible to RNA viruses [17,18]. *OAS2* and *OAS3* recognize dsRNA in different ways [59], which provides an additional means of activating RNase L, potentially reducing *OAS1* activity loss during infection [60]. Take the *OAS1* and *OAS2* genes of *Sus scrofa* as an example. Based on nucleic acid sequence lengths, the sequence of *OAS2* is significantly longer than that of OAS1. After translation into protein, we found that 70% of the amino acids in *OAS1* were similar to those in *OAS2*. We therefore propose the hypothesis that gene duplication occurs in the *OAS1* gene of *Sus scrofa.* This is also consistent with the findings of Perelygin et al. that *OAS1* is genetically duplicated in rodents and even-toed ungulates [58]. The situation should be similar for other species. The replication of different genes may be related to the environment and stresses faced by the species. For example, we noticed that the *OASL* gene of *Capreolus pygargus* was a pseudogene, suggesting that the classic OAS/RNase L signaling pathway is its main antiviral pathway [7]. As a species with high virus resistance, *OAS2* homologous to *OAS1* enhances resistance to viruses.

We observed that the degree of conservation of the OAS gene family varies between species in Laurasiatherian mammals. The ω values of the OASL genes in *Cervidae* and *Giraffidae* were larger than 1. This proves that the genes were subject to positive selection during the evolutionary process. The ω values of the remaining species were less than 1. This indicates that the OAS gene family were also under purifying selection pressure during the evolution of the Laurasiatherian mammals. Thus, the OAS gene family is relatively conservative. The molecular structure of viruses is usually conserved, and the mutations that cause changes in their structures are mostly deleterious. As we mentioned earlier, gene duplication is a general evolutionary mechanism in the OAS gene family [58]. The branch model analysis found that the conservation of the *OAS* genes of *Chiroptera* was higher than that of the other orders. This means that its *OAS* genes are more conserved. *Chiroptera* is a host for many viruses but is not easily infected [61]. Highly conserved genes can better ensure functional stability [62].

Although the *OAS* genes were relatively conserved, positive selection occurred during evolution. We found positive selection sites in both OAS genes of *Cetartiodactyla*. It has been shown in previous studies that the paralogous *OAS1* gene in rodents and cloven-hoofed animals had co-evolved [58]. This also shows that *OAS* genes may have co-evolved with viruses to better resist their threats. Positive selection sites were found in the Felidae and Mustelidae of *Carnivora*. No positive selection sites were found in *Canidae*. Although the OAS genes in *Canidae* are under pressure from purifying selection, the positive selection site deletion indicates poor adaptive evolution of the OAS gene, causing a disadvantage in the arms race with viruses. This may result in *Canidae* species being more susceptible to viruses compared to other species. For example, some studies have shown that *foxes*, *dholes*, *wolves*, *dogs* and other *Canidae* species are extremely vulnerable to rabies virus [18]. Rabies virus is a virus of the genus RNA virus. The OAS gene plays an important role in the fight against this virus [11,63]. Therefore, the absence of positive selection sites may lead to slow adaptive evolution of OAS genes in *Canidae*. This also results in the Canidae being less resistant to rabies virus than other *Carnivora* species. The large number of positively selected sites in the *Cetacea* OAS gene family indicates a high degree of adaptive evolution. This can provide an advantage in the competition with viruses. As a major source genus for domestic animals, a large number of *OAS3* positive selection loci were found for *Equidae.* This suggests that there may be a co-evolutionary process between the host and the virus in an “arms race” to better defend itself against the threat of the virus and pathogenic microorganisms. That is, the adaptive evolution of either species may exert selective pressure on the other, and this interrelationship may drive co-evolution between them [64]. This result strongly supports the “Red Queen Hypothesis” [65]. Some studies have shown that Toll-like receptor 3 (*TLR3*) and the *TLR4*-mediated induction of the genes are modulated by *OAS1* [66]. The activation of RNase L is dependent on *OAS3* expression during infection with diverse human viruses [63], and it can be induced by smaller amounts of double-stranded RNA (dsRNA) [56]. It has been shown that *OAS3* is the most resistant to enteroviruses [67]. Positive sites were also predominantly found in *OAS1* and *OAS3*. This suggests that these two genes may play a more important role in resistance to the virus.

Laurasiatherian mammals have highly specialized anatomy and behavior; for example, they can fly, swim and run quickly, and they are insectivorous and carnivorous [68]. Rabies virus is a common virus of the genus RNA virus, and it is lethal to humans, wild mammals and domestic animals. Research has shown that animals that live together may be more susceptible to viruses than animals that live alone [69]. Herding behavior creates favorable conditions for the spread of rabies virus, resulting in many herd animals being more susceptible to rabies infection. The OAS gene family’s evolution rates were found to be diverse among species, and this implies that different pathogen pressures and habitats influence gene evolution rates [70]. As we mentioned earlier, rabies virus, as a representative RNA virus, poses a great threat to wild mammals. The *Canidae* are also more susceptible to rabies than other families of the *Carnivora*. To address this situation, we have explored three families of *Carnivora.* In our study, no positive selection sites were found in the most rabies-affected *Canids* of the *Carnivora.* Meanwhile, a large number of positive selection sites were found in *Felidae* and *Mustelidae*. OAS gene is a key gene against RNA virus. This suggests that the presence of a positive selection site in the OAS gene plays a role in fight against rabies virus. Overall, species with positive selection sites are more resistant to rabies virus than those without positive selection sites. Of course, the presence of positive selection sites is only part of the mechanism that increases the ability of a species to resist viruses. The rate of gene evolution is also a measure of a species’ ability to resist viruses. The evolution rate of the four genes is also related to the strength of their functions. Adaptive evolution combined with conserved gene function provides improved resistance to viruses. *OAS1* and *OAS3* have stronger functions [58,66,69], and their evolution rates are lower than those of *OAS2*.

## 5. Conclusions

This study provides the first comprehensive analysis of the evolution of the OAS gene family in Laurasiatherian mammals. A total of 238 *OAS* sequences were identified in Laurasiatherian mammals, and this detailed phylogenetic analysis offers a great basis for further functional studies. Our results suggest that the OAS genes are subject to purifying selection pressure in Laurasiatherian mammals, indicating that the OAS gene family has stabilization and significant functional constraints. Different environments put different selection pressures on the OAS genes. The OAS gene family was highly conserved during evolution. A high level of conservatism enables better resistance to viruses. Some *Antilopinae*, *Caprinae* and *Cervidae* species lacked the *OASL* gene, which may be related to their habitats being at higher altitudes. *OASs* had a high number of positive selection sites in Cetartiodactyla, which drove the expression of strong antiviral ability. Except for *OAS1*, in Canidae, the evolution rates of the OAS genes were higher than those in *Feliformia* and *Mustelidae*. Meanwhile, no positive selection sites were found in *Canidae*. This may result in Canidae species with lower resistance to rabies virus. *Chiroptera* is a host for many viruses, and it had the slowest evolutionary rate of the OAS genes. The OAS gene family was relatively conserved during evolution. Conserved genes are able to provide better maintenance of gene function. The positive selection sites demonstrate the adaptive evolution of the OAS gene family to the environment. Adaptive evolution combined with conserved gene function for improved resistance to viruses. *OAS1* and *OAS3* may play more important roles in the fight against viruses. Their exact roles need to be further verified.

## Figures and Tables

**Table 1:** Analysis of positive selection sites for Laurasiatherian mammals species.

*Taxa*	*Gene*	*lnL M7*	*lnL M8*	*No. of Species*	*df*	*p-Values*	*BEB*
*Cetartiodactyla*	*OAS1*	−6072.4755	−6032.329	21	2	3.67 × 10^−18^ **	9 K 0.999 ** 10 S 0.998 ** 26 R 0.967 * 47 C 1.000 ** 110 T 0.951 * 136 A 0.997 ** 189 N 0.999 ** 206 L 0.998 ** 234 K 0.965 * 237 R 0.994 ** 239 N 0.968 * 256 K 0.987 * 263 K 0.975 * 277 T 0.977 * 280 A 0.965 * 302 Y 0.996 ** 312 L 0.969 *
	*OAS2*	−9839.0114	−9832.432	17	2	4.41 × 10^−5^ **	164 H 0.968 * 275 D 0.967 *
	*OAS3*	−7911.3451	−7900.636	17	2	1.07 × 10^−30^ **	7 R 0.999 ** 8 C 1.000 * 11 V 1.000 * 255 G 0.982 * 256 R 0.998 ** 268 G 1.000 ** 275 A 0.999 ** 276 S 1.000 ** 280 L 0.999 ** 468 N 0.954 * 553 R 0.997 **
	*OASL*	−9371.3533	−9371.353	17	2	2.05 × 10^−14^**	106 S 0.996 ** 120 S 0.979 * 143 S 0.985 * 147 F 0.998 ** 160 R 1.000 ** 234 R 0.961 * 241 R 0.984 *
*Pholidota*	*OAS1*	−10,065.963	−10,065.963	−2E-06	2	0.5	
	*OAS2*	−12,224.198	−12,224.198	0	2	0.5	
*Perissodactyla*	*OAS1*	−2693.1406	−2685.5063	4	2	0.000483 **	375 P 0.973 * 378 T 0.995 **
	*OAS2*	−4982.3562	−4983.0484	4	2	0.500476317	
	*OAS3*	−7739.7452	−7740.2327	4	2	0.614127	
	*OASL*	−1712.6166	−1715.0368	4	2	0.5	
*Carnivora*	*OAS1*	−7562.7926	−7593.4765	26	2	4.72 × 10^−14^ **	11 Y 0.980 * 158 G 0.987 * 161 N 0.969 * 163 E 0.982 * 167 T 0.995 ** 170 R 0.997 ** 173 Q 0.978 * 189 Q 0.978 * 273 G 0.992 **
	*OAS2*	−11,635.337	−11,649.541	25	2	6.77 × 10^−7^ **	188 H 0.985 * 213 L 0.965 * 215 H 0.989 * 368 E 0.984 * 376 R 0.971 *
	*OAS3*	−20,393.582	−20,496.004	25	2	3.30 × 10^−45^ **	1 D 0.990 ** 2 S 0.997 ** 4 V 0.999 ** 6 R 0.996 ** 7 N 0.998 ** 8 L 0.986 ** 9 M 0.980 * 11 S 0.999 ** 13 L 0.993 ** 14 A 0.959 * 15 A 0.999 ** 24 A 0.997 ** 36 K 0.995 ** 69 R 0.996 ** 92 H 0.961 * 136 S 0.995 * 172 T 0.987 * 189 C 1.000 ** 260 R 0.988 * 335 S 0.998 ** 348 T 0.978 * 407 S 0.996 ** 520 S 0.980 *
	*OASL*	−7234.9379	−7242.5053	26	2	0.0005169 **	390 H 0.977 *
*Chiroptera*	*OAS1*	−4036.7745	−4047.5839	5	2	2.02 × 10^−5^ **	4 E 0.954 * 231 T 0.968 *
	*OAS2*	−3866.3837	−3866.5936	5	2	0.810632	
	*OAS3*	−10,106.391	−10,113.077	5	2	0.0012473 **	
	*OASL*	−6080.1380	−6084.3458	5	2	0.0148787 **	
*Eulipotyphla*	*OAS1*	−4114.7990	−4124.5812	6	2	5.64 × 10^−5^ **	153 L 0.95 9 * 154 R 0.977 * 212 P 0.967 * 234 D 0.966 *
	*OAS2*	−6875.2726	−6896.9948	6	2	3.68 × 10^−10^ **	144 K 0.962 * 162 E 0.988 * 189 S 0.980 * 253 N 0.975 * 310 R 0.979 *
	*OAS3*	−12,026.957	−12,033.766	6	2	0.0011045 *	
	*OASL*	−2818.2825	−2821.8173	6	2	0.02916485 *	

* The significant level ** (*p* < 0.01); * means Bayes Empirical Bayes (BEB) > 0.95; ** means BEB > 0.99.

**Table 2:** Positive selection sites obtained from the FUBAR model.

Taxa	Gene	Positive Selection (0.9)	Positive Selection (0.95)
*Cetartiodactyla*	*OAS1*	27	14
	*OAS2*	4	1
	*OAS3*	6	1
	*OASL*	6	1
*Perissodactyla*	*OAS1*	1	1
	*OAS2*	4	0
	*OAS3*	0	0
	*OASL*	3	1
*Carnivora*	*OAS1*	1	0
	*OAS2*	1	1
	*OAS3*	2	0
	*OASL*	1	1
*Chiroptera*	*OAS1*	4	1
	*OAS2*	2	0
	*OAS3*	2	0
	*OASL*	0	0
*Eulipotyphla*	*OAS1*	1	0
	*OAS2*	3	0
	*OAS3*	2	0
	*OASL*	3	0

**Table 3:** Tests for positive selection in different clades of OAS genes with a branching model.

Taxa	Gene	lnL M0	lnL M1	*p*-Values	ω0	ω	Order
*Carnivora*	*OAS1*	*−7886.75*	*−7885.87*	*0.9393*	*0.37587*	*0.42983* *0.32010* *0.35686* *0.32953* *0.41482* *0.29480*	*Mustelidae* *Canidae* *Feliformia* *Ailuridae* *pinnipedia* *Ursidae*
	*OAS2*	*−11,883.4*	*−11,867.53*	1.75E-05 **	*0.33957*	*0.44169* *0.50012* *0.30951* *0.36258* *0.55048* *0.20961*	*Mustelidae* *Canidae* *Feliformia* *Ailuridae* *pinnipedia* *Ursidae*
	*OAS3*	*−21,140.7*	*−21,130.42*	0.00218 **	*0.31004*	*0.23408* *0.46858* *0.33034* *0.34092* *0.34686* *0.83956*	*Mustelidae* *Canidae* *Feliformia* *Ailuridae* *pinnipedia* *Ursidae*
	*OASL*	*−7315.46*	*−7311.68*	*0.27016*	*0.3606*	*0.34322* *0.37139* *0.26894* *0.49875* *0.60788* *0.40809*	*Mustelidae* *Canidae* *Feliformia* *Ailuridae* *pinnipedia* *Ursidae*
*Eulipotyphla*	*OAS1*	*−4257.98*	*−4256.004*	*0.4122*	*0.2864*	*0.29963* *0.32943* *0.32723* *0.18820*	*Talpidae* *Erinaceidae* *Solenodontidae* *Soricidea*
	*OAS2*	*−7181.19*	*−7140.64*	1.01E-16 **	*0.20656*	*0.45990* *0.11458* *0.16019* *0.03739*	*Talpidae* *Erinaceidae* *Solenodontidae* *Soricidea*
	*OAS3*	*−12,355.6*	*−12,349.08*	0.01026 *	*0.19660*	*0.19437* *0.16947* *0.20071* *0.14918*	*Talpidae* *Erinaceidae* *Solenodontidae* *Soricidea*
	*OASL*	*−2867.49*	*−2863.16*	*0.0737*	*0.34407*	*0.29138* *0.34153* *0.77844* *0.26496*	*Talpidae* *Erinaceidae* *Solenodontidae* *Soricidea*
*Chiroptera*	*OAS1*	*−4183.03*	*−4164.62*	6.48E-07 **	*0.24726*	*0.27718* *0.35271* *0.38371* *0.27003* *0.00970*	*Rhinolophidae* *Vespertilionidae* *Molossidae* *Pteropodidae* *Nycteridae*
	*OAS2*	*−3940.66*	*−3937.56*	*0.2871*	*2.48390*	*0.15611* *0.14157* *0.14517* *0.12003* *0.50989*	*Rhinolophidae* *Vespertilionidae* *Molossidae* *Pteropodidae* *Nycteridae*
	*OAS3*	*−10,250.7*	*−10,247.75*	*0.3047*	*0.30613*	*0.23936* *0.33791* *0.26711* *0.35137* *0.30723*	*Rhinolophidae* *Vespertilionidae* *Molossidae* *Pteropodidae* *Nycteridae*
	*OASL*	*−6204.10*	*−6193.65*	*0.3047*	*0.27926*	*0.23185* *0.31819* *0.43633* *0.17294* *0.05074*	*Rhinolophidae* *Vespertilionidae* *Molossidae* *Pteropodidae* *Nycteridae*
*Cetartiodactyla*	*OAS1*	*−6270.46*	*−6264.38*	*0.14158*	*0.49856*	*0.27123* *0.52040* *0.28835* *0.31889* *0.84189* *0.65807* *0.37722* *0.41818*	*Moschidae* *Bovidae* *Cervidae* *Giraffidae* *Hippopotamidae* *Odontoceti* *Camelidae* *Suidae*
	*OAS2*	*−4926.82*	*−4921.97*	*0.258364*	*0.467758*	*0.66480* *0.52956* *0.74130* *0.44746* *0.48362* *0.66494* *0.45639* *0.33798*	*Moschidae* *Bovidae* *Cervidae* *Giraffidae* *Hippopotamidae* *Odontoceti* *Camelidae* *Suidae*
	*OAS3*	*−8328.28*	*−8317.61*	1.32E-05 **	*0.426209*	*0.26979* *0.59390* *0.38201* *0.80675* *0.50011* *0.36829* *0.31322* *0.32099*	*Moschidae* *Bovidae* *Cervidae* *Giraffidae* *Hippopotamidae* *Odontoceti* *Camelidae* *Suidae*
	*OASL*	*−5154.22*	*−5126.18*	2.72E-09 **	*0.50055*	*0.02728* *0.71554* *1.02181* *1.03467* *0.71635* *0.40102* *0.46758* *0.67712*	*Moschidae* *Bovidae* *Cervidae* *Giraffidae* *Hippopotamidae* *Odontoceti* *Camelidae* *Suidae*
*Perissodactyla*	*OAS1*	*−2705.67*	*−2699.96*	0.0097 **	*0.37987*	*0.68667* *0.63466* *0.44241*	*Equidae* *Rhinocerotidae* *Tapiridae*
	*OAS2*	*−5009.23*	*−5007.79*	*0.4109*	*0.40647*	*0.22805* *0.45730* *0.32208*	*Equidae* *Rhinocerotidae* *Tapiridae*
	*OAS3*	*−7799.66*	*−7796.08*	*0.0669*	*0.2828*	*0.37966* *0.29006* *0.27405*	*Equidae* *Rhinocerotidae* *Tapiridae*
	*OASL*	*−1722.41*	*−1722.147*	*0.9124*	*0.2445*	*0.31200* *0.26917* *0.19742*	*Equidae* *Rhinocerotidae* *Tapiridae*

* The significant level: * (*p* < 0.05); ** (*p* < 0.01).

## Data Availability

All the genomes’ sequences used in this study were accessed through the GenBank database using the accession numbers in Table S1. The branch point model data is in Table S2.

## Supplementary Materials

The following supporting information can be downloaded at: https://www.mdpi.com/article/10.3390/ani13020209/s1, Table S1: Genomic data used in experiments. Table S2: Positive selection for OAS gene branch locus models in Laurasiatherian mammals.
