# Supplementary material for: Adaptive Evolution of the OAS Gene Family Provides New Insights into the Antiviral Ability of Laurasiatherian Mammals
Source: Animals (Basel). 2023 Jan 6;13(2):209. doi: 10.3390/ani13020209 (PMC9854896; doi:10.3390/ani13020209)
Supplement: Supplementary file 1 [file animals-13-00209-s001.zip › Table S1.pdf]

Species Name	Genome Assembly Number	Contig N50 (bp)	Depth (×)
Condylura cristata	GCA_000385615.1	46,163	113.1
Erinaceus europaeus	GCA_000296755.1	21,359	79
Scalopus aquaticus	GCA_003846335.1	72,421	76.1
Solenodon paradoxus	GCA_004363575.1	236,847	29.3
Sorex araneus	GCA_000181275.2	22,623	120
Uropsilus gracilis	GCA_004024945.1	43,618	54.2
Rhinolophus ferrumequinum	GCA_014108255.1	21,749,602	73.3
Murina aurata	GCA_004026665.1	23,400	33.3
Molossus molossus	GCA_014108415.1	22,174,888	58.3
Rousettus leschenaultii	GCA_015472975.1	40477	308
Craseonycteris thonglongyai	GCA_004027555.1	23218	52.2
Giraffa camelopardalis	GCA_017591445.1	11,323,477	54
Elaphurus davidianus	GCA_021018665.1	32707	82
Capreolus pygargus	GCA_012922965.1	80,310	100
Procapra przewalskii	GCA_006410515.1	10,405	209
Moschus moschiferus	GCA_004024705.2	34,785	74.6
Moschus chrysogaster	GCA_006461725.1	3,769	310
Pseudois nayaur	GCA_003182575.1	20,979	288
Ovis canadensis	GCA_004026945.1	55,973	25.2
Bison bison	GCA_000754665.1	19,971	60.0
Bos grunniens	GCA_005887515.2	44,716,738	239.0
Camelus ferus	GCA_009834535.1	5,365,398	50.0

Connochaetes taurinus	GCA_006408615.1	70,608	216
Hippopotamus amphibius	GCA_004027065.2	76,609	77
Sus scrofa	GCA_000003025.6	48,231,277	65.0
Neophocaena asiaeorientalis	GCA_003031525.2	86,003	106
Sousa chinensis	GCA_007760645.1	113,766	283.0
Platanista gangetica	GCA_017311385.1	3,092	10.0
Lipotes vexillifer	GCA_000442215.1	31,902	115
Orcinus orca	GCA_000331955.2	70,300	200.0
Physeter catodon	GCA_002837175.2	42,542	248
Eschrichtius robustus	GCA_002189225.1	10,066	11.0
Equus przewalskii	GCA_000696695.1	57,610	85.63
Equus asinus	GCA_016077325.2	25,073,460	211.0
Dicerorhinus sumatrensis	GCA_014189135.1	70,892	38.0
Tapirus indicus	GCA_004024905.1	225,792	41.4
Lontra canadensis	GCA_010015895.1	691,969	40.0
Enhydra lutris	GCA_002288905.2	244,529	110.0
Neovison vison	GCA_020171115.1	220,349,31	21.0
Martes zibellina	GCA_012583365.1	62,196	114.0
Nyctereutes procyonoides	GCA_905146905.1	35,077,230	106
Vulpes lagopus	GCA_018345385.1	33,460,300	121.0
Vulpes vulpes	GCA_003160815.1	55,450	93.9
Ailurus fulgens	GCA_002007465.1	99,577	115.5
Suricata suricatta	GCA_006229205.1	75,409	50.0

Lynx pardinus	GCA_900661375.1	99,542	135
Felis catus	GCA_018350175.1	90,731,473	76.0
Canis lupus familiaris	GCA_014441545.1	12,024,593	56.5
canis lupus	GCA_014441545.1	12,024,593	56.5
Hyaena hyaena	GCA_003009895.1	311,202	56.0
Callorhinus ursinus	GCA_003265705.1	133,024	27.44
Mirounga angustirostris	GCA_021288785.2	76,189	125.0
Mustela erminea	GCA_009829155.1	36,329,944	62.86
Mellivora capensis	GCA_004024625.1	50,213	60
Taxidea taxus	GCA_003697995.1	37,750	44.0
Panthera pardus	GCA_001857705.1	20,993	158.5
Panthera onca	GCA_004023805.1	62,836	60.7
Panthera leo	GCA_018350215.1	77,781,637	76.0
Panthera tigris	GCA_018350195.2	74,391,967	78.0
Ailuropoda melanoleuca	GCA_002007445.2	127,363	93.0
Ursus arctos	GCA_003584765.2	314,844	50.0
Ursus maritimus	GCA_017311325.1	206,653	52.0
Manis pentadactyla	GCA_014570555.1	151,937	281.6
Manis crassicaudata	GCA_016801295.1	7,447	44.0
