# Supplementary material for: Adaptive Evolution of the OAS Gene Family Provides New Insights into the Antiviral Ability of Laurasiatherian Mammals
Source: Animals (Basel). 2023 Jan 6;13(2):209. doi: 10.3390/ani13020209 (PMC9854896; doi:10.3390/ani13020209)
Supplement: Supplementary file 1 [file animals-13-00209-s001.zip › Table S2.pdf]

Table S2. Positive selection for OAS gene branch locus models in Laurasiatherian mammals.

Order	Family	Gene	lnL M1a	lnL M2a	LRT	df	*P*-values	BEB
		OSA1	-6047.98	-6058.65	21.34458	1	3.84E-06**	155 Y 0.986*<br>174 E 0.980*<br>256 K 0.977*
		OAS2	-12172.076	-12166.514	4E-05	1	0.9445	
Cetartiodactyla	*Cetacea*	*OAS3*	-14365.6	-14429.7	128.1	1	1.03E-29**	2 F 0.994**<br>3 L 0.953*<br>4 K 0.950*<br>7 R 1.000**<br>11 V 0.995**<br>268 G 0.959*<br>276 S 1.000**
		OASL	-5063.2474	-5063.2474	0	1	1	
		OAS1	-6063.95	-6063.9423	0	1	1	
		OAS2	-4817.4778	-4819.3888	3.822	1	0.050583	
	Ruminantia	*OAS3*	-14418.27	-14428.84	21.12321	1	4.31E-06**	8 C 0.978*<br>553 R 0.989*
		OASL	-5060.514	-5061.242	1.4563	1	0.2275	
		OSA1	-2688.1805	-2691.3826	6.40423	1	0.011385	
		OAS2	-4982.758	-4982.758	0.01	1	0.974773	
Perissodactyla	*Equidae*	*OAS3*	-7649.3323	-7734.5040	170.3434	1	6.23E-39**	282 T 0.979*<br>283 H 0.972*<br>289 R 0.964*<br>449 A 0.980*<br>450 P 0.979*<br>451 M 0.998**<br>452 G 0.973*<br>464 K 0.970*<br>488 S 0.986*<br>490 K 0.976*<br>492 E 0.997**<br>495 R 0.965*<br>502 K 0.969*<br>508 Q 0.958*<br>513 F 0.969*<br>514 E 0.977*<br>516 K 0.965*<br>529 S 0.996**<br>531 S 0.993**<br>539 E 0.997**<br>544 D 0.974*
		OASL	-1714.903	-1714.903	0	1	1	
		OSA1	-7594.9281	-7594.9281	0..000178	1	0.989355	
		OAS2	-11799.924	-11799.924	0	1	1	
	Canidae	*OAS3*	-20526	-20526.8	1.5625	1	0.2113	
Carnivora		*OASL*	-7237.6217	-7237.621	1E-05	1	0.9974	
	Felidae	*OSA1*	-7564.17	-7574.90	21.458	1	3.62E-06**	165 Y 0.999**<br>179 F 0.998**<br>181 E 0.999**<br>186 F 0.996**

								196 L 0.995**<br>202 H 0.985*
		OAS2	-11823.7	-11823.7	0.0374	1	0.846	
		OAS3	-20437.42	-20528.03	181.2367	1	2.6E-41**	1 D 0.999**<br>3 L 0.992**<br>4 V 1.000**<br>6 R 0.993**<br>7 N 0.997**<br>8 L 0.999**<br>424 S 0.998**<br>480 L 0.971*
		OASL	-7837.621	-7837.621	0	1	1	
		OAS1	-7594.9281	-7594.93	4E-06	1	0.9984	
		OAS2	-11820.9905	--11811.7383	18.5043	1	1.7E-05	
	Mustelidae	*OAS3*	-20467.8822	-20465.8822	4.0046	1	0.04548*	191 V 0.978*<br>195 E 0.951*<br>225 S 0.973*<br>306 Q 0.971*<br>317 G 0.979*<br>329 H 0.983*<br>330 P 0.981*<br>336 A 0.983*<br>367 G 0.960*<br>378 K 0.977*<br>385 Q 0.981*<br>387 H 0.986*<br>396 K 0.973*<br>408 C 0.979*<br>420 V 0.983*<br>421 S 0.965*<br>454 A 0.975*<br>473 D 0.955*<br>486 T 0.972*<br>502 S 0.981*<br>1004 S 0.983*
		OASL	-7237.20	-7327.20	0	1	1	
		OSA1	-4043.34	-4047.19	7.698844	1	0.00552**	
Chiroptera	*Vespertilionidae*	*OAS2*	-3863.3988	-3872.2827	17.76763	1	2.5E-05**	2 L 0.988*<br>16 S 0.972*<br>38 S 0.984*
		OAS3	-10116.494	-10118.108	3.227	1	0.07241	
		OASL	-6082.457	-6085.728	6.5425	1	0.0105*	
		OSA1	-4116.178	-4123.397	14.4328	1	0.000145**	168 E 0.975*
Eulipotyphla	*Talpidae*	*OAS2*	-6841.149	-6861.554	40.8109	1	1.68E-10**	42 K 0.996**<br>61 K 0.956*<br>88 E 0.958*<br>93 S 0.954*<br>189 S 0.980*<br>204 R 0.989*<br>225 T 0.965*<br>235 L 0.973*<br>237 A 0.994**<br>238 M 0.961*<br>263 R 0.973*

295 R 0.957*
312 G 0.988*
319 V 0.991**

OAS3	-12066.599	--12066.599	0.0016	1	0.968
OASL	-2823.6052	-2823.6862	0.162	1	0.687322

Notes: * The significant level :*（$0.01 < p < 0.05$）,**（$p < 0.01$）
